# Special libraries, special challenges: An ethical framework for access to problematic historical medical films

**DOI:** 10.1080/15228959.2025.2455216

**Published:** 2025-02-02

**Authors:** Katie Lattal, Mohammad Hosseini, Kristi Holmes

**Affiliations:** a Galter Health Sciences Library and Learning Center, Northwestern University Feinberg School of Medicine, Chicago, IL, USA;; b Department of Preventive Medicine, Northwestern University Feinberg School of Medicine, USA

**Keywords:** Ethics, access, privacy, digitization, archives, film preservation, audiovisual archives

## Abstract

Medical films, once a staple of medical education in the twentieth century, are now scarce collection items. Many 16 mm films have been weeded from collections because of their outdated content and obsolete format, and those that remain can suffer rapid deterioration from improper storage conditions. Digitization is imperative to preserve these remnants of our cultural heritage. After digitization is complete and rights are cleared, libraries typically make their collections openly accessible online, but this practice is sometimes complicated due to the content of historical medical films. Without a mechanism to regulate patron access to films with potentially problematic content, libraries must tightly restrict these newly preserved resources. This paper presents a framework that defines problematic content based on four criteria and proposes a tiered approach to access.

## The indispensability of preservation

Medical motion pictures (hereafter medical films) are unique resources straddling the humanities and health sciences. Medical films are distinct from public health and other educational films in that they are designed for medical professionals and students instead of the general public. These films capture and convey scientific, medical, cultural, visual, and personal information through a medium that engages multiple senses and allows for a large amount of visual (and sometimes spoken) information to be conveyed quickly to the viewer. Medical films are notable for their interdisciplinary value, as they are primary sources for research and teaching in fields as varied as the health sciences, media, history, medical humanities, anthropology, and education.

There are few collections of historical medical films accessible and discoverable today, and those that have survived in archives are scattered, lying in “long forgotten corners and cupboards,” resulting in incomplete documentation and limited accessibility (Tansey, 1993). The National Library of Medicine’s (NLM) audiovisual collection—curated and contextualized in the *Medicine on Screen* portal—is a noteworthy exception of publicly available medical films (NLM, n.d.). Beyond NLM, sizeable collections of historical medical films are rare in the U.S.,^1^ and even locating medical films is difficult (Dong et al., 2018). Outdated or unpopular media formats are often under-described or uncatalogued and therefore hidden from users (Gracy & Kahn, 2012). Against this backdrop, explorations like Imre and Cox’s study of LP collections in U.S. academic libraries is relevant because of the LP’s similar status as a format considered obsolete for academic research libraries. This study found that 25% of LPs in academic libraries were uncatalogued, and highlighted lack of bibliographic control as the biggest access barrier. Furthermore, since collections of obsolete media formats are hidden and therefore underutilized, it can be difficult to justify retention (Imre & Cox, 2009).

Of the thousands of medical films that once circulated across the U.S., it is likely that many copies were weeded out of institutional collections for a variety of reasons (Dong et al., 2018). For example, when VHS and later DVD formats overtook 16 mm films, most libraries prioritized these cheaper formats. Accordingly, bulky films were deaccessioned to create collections space and “even rare out-of-print materials were deaccessioned if the informational content was seen as outdated (Gracy, 2013).”

The Association of College and Research Libraries (ACRL) Code of Ethics for Special Collections Librarians and the Society of American Archivists (SAA) Core Values Statement and Code of Ethics both include preservation as a key tenet of the profession, stating that “preservation of collections [is] a fundamental responsibility of stewardship” (ACRL, 2020) and that preservation is the means by which future accessibility is possible (Society of American Archivists [SAA], 2005).

While the rarity of historical medical films compels us to preserve them, it is the instability of the physical media and aging playback equipment that makes preservation necessary and urgent. 16 mm films were mostly shot on nitrate, acetate, and polyester film stock. The instability of nitrate and acetate films can result in rapid decay even if stored in improper conditions for short periods of time (Gracy & Kahn, 2012; Poole, 1999). Historical medical films should be assessed for digitization since, given the age of the substrate, it is likely that some deterioration or loss has already occurred. However, the digitization of medical films presents challenges for many special libraries and archives, whose small staff may not include a digital archivist, preservation librarian, or even a staff member with audio/video (AV) expertise. AV digitization projects involve specialist knowledge of physical and digital formats, staff time and resources for description and metadata, cost of digitization vendors, and large amounts of physical and digital storage space. Furthermore, after digitization is complete, a digital preservation plan that details the required technical systems and infrastructure, condition monitoring protocols, and plans for media and format migration must be implemented for responsible stewardship (IASA Technical Committee, 2017).

## Managing access to problematic medical films

Providing access to historical medical films is becoming more challenging as more researchers expect content to be openly accessible online. But providing free and open access to some films, which, for instance, may display subjects whose conditions of consent are unknown, may inflict further harm on the subjects. Indeed, despite the significance of preserving medical films for future medical, historical, and cultural analyses, not all films are appropriate to be widely shared (Beauchamp & Childress, 2009). One of the key features of the norms of science (i.e., values, virtues, and principles related to the conduct of science) is that they evolve over time (Hosseini et al., 2024). In other words, what was considered acceptable in the beginning of the twentieth century might be completely inappropriate now. For example, at the end of the nineteenth century and start of the twentieth century, many scientists supported eugenics, but after World War II, they distanced themselves from it (National Human Genome Research Institute [NHGRI], 2021). Some of these altered norms are relevant to medical films, especially concerning privacy, confidentiality, and consent. For example, some films may involve innocuous scenes, like a patient smiling at the camera to display a well-healed facial scar post-surgery, or a patient describing their condition ahead of treatment. In the absence of consent forms, these films are privacy-breaching by today’s standards. Other films may feature graphic scenes that should not be in the public domain because of ethical considerations or concern for their impact on viewers’ mental health, such as films depicting surgical obstetric interventions or pediatric patients. Addressing these issues can add another layer of complexity to under-resourced special libraries.

The Special Collections Department at Galter Health Sciences Library and Learning Center (hereafter Galter Library) developed a reusable framework for managing access to problematic medical films that defines access to problematic content in a nuanced way so that patron access could be maximized while supporting ethical standards. Such a framework would be particularly useful in contexts where redaction is impractical, for example, because it would render edited films less useful and diminish their research value (Letocha, 2009). Our first challenges were how to define *problematic*, and how to identify and regulate access levels. In what follows, we explain how these challenges were addressed.

Over the course of many months, we consulted subject experts in law, medical humanities, bioethics, and archives, who all advised some level of risk-averse approach and highlighted specific issues to consider. Next, we searched the literature to find examples of libraries that had dealt with similar challenges. After reviewing the Health Insurance Portability and Accountability Act (HIPAA) Resource Page (ALHHS, 2014),^2^ we searched PubMed (on 22 January 2024) for “ethics” OR “privacy” AND “historical research” and the MeSH term “Libraries, Medical/ethics,” both of which returned less than ten results without fully addressing the range of ethical concerns related to our collection. Searches for the MeSH term “Health Insurance Portability and Accountability Act” accompanied by multiple secondary search filters (e.g., history; historical; historical research) returned more results, but the vast majority did not pertain to the *ethics* of access to historical collections. These searches were repeated in Galter Library’s catalog and netted a few more resources but none were directly relevant to medical films. Of note, since HIPAA was updated in 2013, it has affected the current applicability of numerous papers published before then (Lawrence, 2007; Wiener & Gilliland, 2011). Gustainis and Letocha’s report (2015) was particularly helpful for recommendations regarding access to collections that contain PHI. However, here too, ethical questions about access to graphic content and explicit images of medical films remained unexplored. Ultimately, we did not find clear guidance or consensus regarding access and use restrictions for problematic medical films.

## A framework to define problematic content

After researching the literature and consulting with the subject experts, we sought feedback from library staff and invited them to several brainstorming sessions about developing an exploratory framework to define problematic content and, accordingly, access levels. Then, the authors and staff tallied various ethical issues discussed during the brainstorming sessions and compared these with findings from the literature search to compile a list of ethical issues. Once we agreed on a final list, we used an inductive approach to formulate a generalizable definition for problematic content. This process involved subsuming similar issues (e.g., expression of pain), or those that touched on similar core principles (e.g., privacy), under a specific category. We then refined these categories in discussions and reached consensus about our definitions. Subsequently, we agreed that problematic content refers to any medical film that includes at least one of the criteria in Table 1.

**Figure 1.**
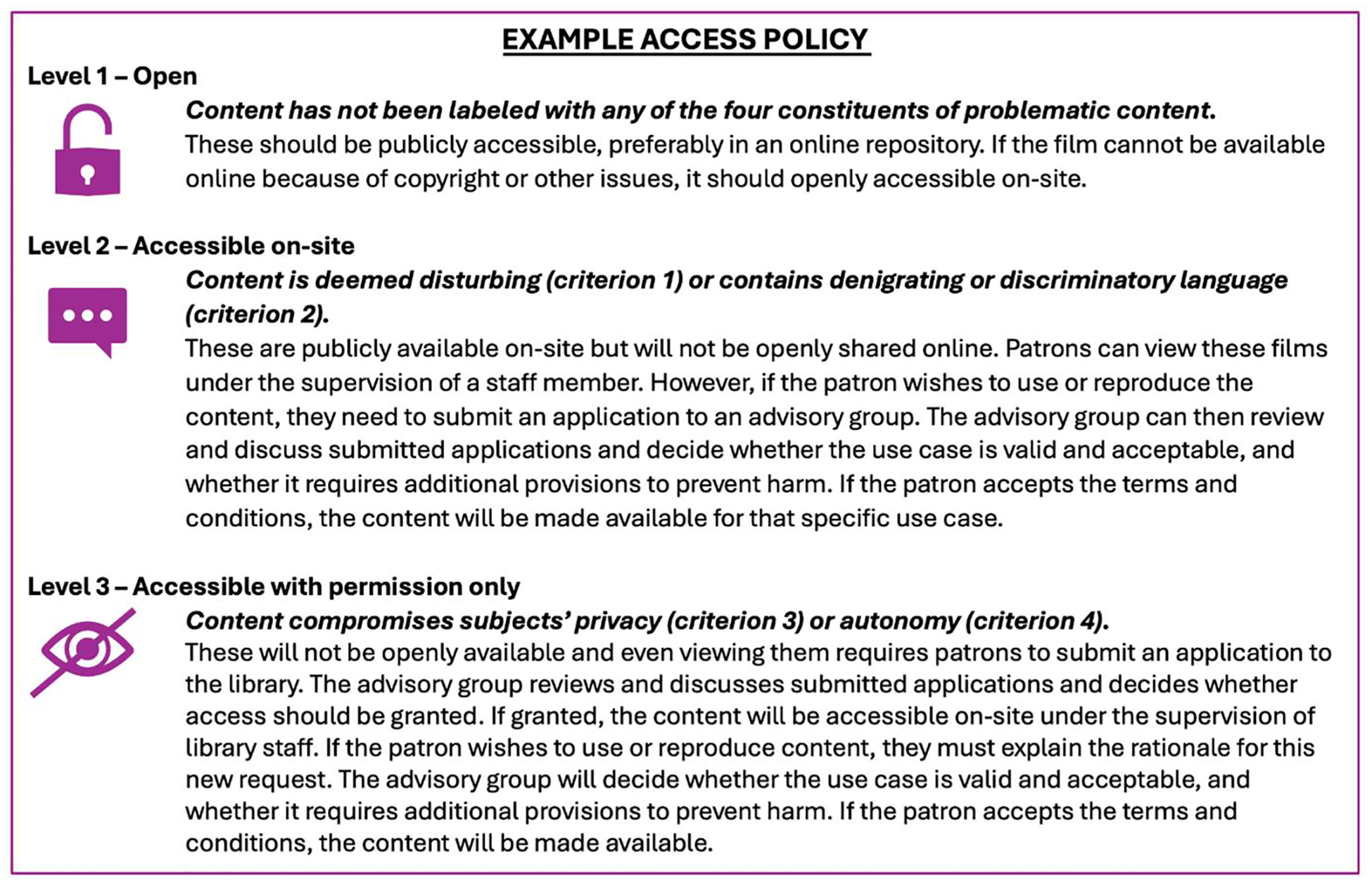
Example Access Policy applying the framework.

Based on this framework, different policies could be developed to determine under what circumstances users could access or use films. Different libraries can use this framework to define access levels based on their available resources, context, and content. An advisory group can help guide any decisions about access. Such a group should (ideally) consist of both library and non-library staff with expertise related to historical medical film collections, such as (bio)ethics, medical humanities, film studies, archives, law, etc. This group must be knowledgeable of HIPAA and privacy regulations so it can take these legal standards into consideration when making decisions on applications for access and use of content. An example access policy that includes three access levels and involves an advisory group is shown in Figure 1.

## Conclusion and next steps

The framework introduced in this paper aims to improve access to historical medical film collections without perpetuating harm that may have been inflicted in the creation of these films. It provides a systematic approach to rating content by considering the ethical implications of access. Judging an item within the context of its creation and the current norms allows us to ethically provide access to–and facilitate the use of–problematic content beyond an assessment of HIPAA or privacy law violations. In addition, creating an advisory group to use the developed framework ensures a collective and balanced decision-making process, instead of relying on *ad hoc* decisions made by one individual librarian.

There is more to do to further develop this model of access. HIPAA remains vague regarding historical medical records, hampering research and leaving library and archives staff in a tough position between users and institutional policies (Letocha, 2013). Notably, this framework can continue to evolve to meet the needs of current and future collections as well as users. We hope that by communicating this framework with the wider community, we can inspire further discussions on this topic by others who may be challenged by similar issues, especially those working with historical medical collections or other specialized libraries.

## Figures and Tables

**Table 1.** Criteria to determine problematic content in historical medical films.

Disturbing content	Visible or audible expressions of pain Loss of limb(s) or life Cruelty against animals Footage of incidents that led to injuries
Denigrating or discriminatory language	Disrespectful or humiliating terms against individuals or groups, including but not limited to those that have sexist, racist, homophobic, ageist, ableist, xenophobic, religious intolerance, ethnic slurs, body shaming, and classist connotations Outdated or insensitive use of terms related to mental or physical conditions (e.g., crippled)
Compromising subjects’ privacy	Subjects’ faces, voices, names, or any of the eighteen PHI identifiers delineated in HIPAA
Compromising subjects’ autonomy	Persons who did not have the capacity for consent at the time of recording, e.g., patients with mental disability or dementia Subjects who could not have consented at the time of recording, e.g., those who were unconscious or under the influence of psychoactive substances upon admission Prisoners, subjects under duress, or those expressing a desire not to be part of the experiment, operation, or recording

## Footnotes

1. Of the four extramural film collections listed under the Resources tab of NLM’s *Medicine on Screen* portal, MedFilm and the Wellcome Collection are based in Europe, Atomic Theater exclusively holds public health films related to atomic attack, and a small number of medical films are featured in the Library of Congress’s National Screening Room.

2. The HIPAA Resource Page was created by Archivists and Librarians in the History of the Health Sciences (ALHHS), which changed its name to Librarians, Archivists, and Museum Professionals in the History of the Health Sciences (LAMPHHS) in 2020. LAMPHHS has updated this page with more resources since our initial review in 2021 (https://lamphhs.org/resources/). You can find the 2014 version of the page via the Wayback Machine: https://web.archive.org/web/20210106200041/ http://iis-exhibits.library.ucla.edu/alhhs/.
